# Reconstruction of the domain orientation distribution function of polycrystalline PZT ceramics using vector piezoresponse force microscopy

**DOI:** 10.1038/s41598-017-18843-4

**Published:** 2018-01-11

**Authors:** Markus Kratzer, Michael Lasnik, Sören Röhrig, Christian Teichert, Marco Deluca

**Affiliations:** 1 Institute of Physics, Montanuniversität Leoben, Franz Josef Straße 18, 8700 Leoben, Austria; 2 Materials Center Leoben Forschung GmbH, Roseggerstraße 12, Leoben, Austria; 3 Institut für Struktur- und Funktionskeramik, Montanuniversität Leoben, Peter Tunner Straße 5, 8700 Leoben, Austria; 4 Present Address: voestalpine Schienen GmbH, Kerpelystraße 199, 8700 Leoben/Donawitz, Austria

## Abstract

Lead zirconate titanate (PZT) is one of the prominent materials used in polycrystalline piezoelectric devices. Since the ferroelectric domain orientation is the most important parameter affecting the electromechanical performance, analyzing the domain orientation distribution is of great importance for the development and understanding of improved piezoceramic devices. Here, vector piezoresponse force microscopy (vector-PFM) has been applied in order to reconstruct the ferroelectric domain orientation distribution function of polished sections of device-ready polycrystalline lead zirconate titanate (PZT) material. A measurement procedure and a computer program based on the software Mathematica have been developed to automatically evaluate the vector-PFM data for reconstructing the domain orientation function. The method is tested on differently in-plane and out-of-plane poled PZT samples, and the results reveal the expected domain patterns and allow determination of the polarization orientation distribution function at high accuracy.

## Introduction

Lead zirconate titanate (PZT) material is one of the most widely used piezoelelectric materials in state-of-the-art piezoelectric devices like actuators^1,2^, transducers^3^, and sensors^4,5^. Its wide application is justified by its high piezoelectric response (large-field d_33_ > 600 pm/V^6^) and high degree of control on the ceramic fabrication process. PZT and other ferroelectric materials exhibit a spontaneous polarization below the Curie temperature (for PZT 500 K–770 K, depending on Ti content). The polarization direction can be altered when a sufficiently large electric field is applied (poling). The polarization direction aligns with the direction of the applied field and persists when the external field is removed (remanent state). Such poled materials exhibit macroscopic piezoelectric behavior. The PZT technology is mainly based on polycrystalline material, since the fabrication of PZT(poly) is technologically much easier to handle than the controlled growth of large single crystals. However, there are some drawbacks associated with the polycrystallinity. In polycrystalline materials, the crystallographic orientation of the grains is essentially random, which also leads to a random orientation of the piezoelectric domains prior to poling. Disadvantageous grain size distribution and domain orientation can severely limit the device performance. The major performance parameter is the large-field constant d_33_ or - in other words - the obtainable magnitude of the electric field induced elongation (strain). Further characteristic values concern the stability, creep, and hysteresis of the system. All these parameters are crucially influenced by the domain distribution in the PZT material. To gain control over the domain distribution it is of utmost importance to obtain knowledge on the domain distribution function. For this, a reliable characterization technique is needed. So far, methods like X-ray and neutron diffraction, electron diffraction (EBSD), or Raman spectroscopy are the widely used state of the art techniques^7,8^. An atomic force microscopy (AFM) based technique - established in the last two decades as an appropriate method for the non-destructive nanometer scale imaging/characterization - is so called piezo response force microscopy (PFM)^9–15^. The advantage of PFM is, in principle, the capability to determine the exact direction (polarity) of the polarization vector, an aspect that is inaccessible to the other techniques mentioned before.

PFM relies on the deformation of the surface due to the converse piezoelectric effect in the electric field of a biased conductive AFM probe tip which is scanned in contact mode across the surface. Since the surface response will in general be very small (few to several 10 pm), a lock–in technique^15^ is used to read it out. For that purpose, an AC voltage is applied to the tip or the sample (frequency for standard PFM typically ƒ ~ 10–100 kHz, amplitude *V*_0_ ~1 – 10 V) resulting in an alternating field under the tip which induces a piezo-mechanic response of the same frequency. The tip follows the tiny surface displacement resulting in a cantilever deflection at the excitation frequency which is read out via standard AFM feedback control. The total response signal contains the magnitude of the deformation (amplitude) and the phase shift with respect to the excitation voltage (phase). While the amplitude varies with relative domain orientation with respect to the electric field, applied voltage, piezoelectric coefficient, etc., the phase response must either be in phase (0°) or out of phase (180°) regarding to the excitation voltage^16^. Deviating phase values originate from inherent system background and electronic delay within the electronics of the setup^17^. Evaluation of the periodic cantilever deflection just provides the normal component of the tip displacement associated with the out-of-plane component of the polarization vector and is usually referred to as vertical PFM (VPFM)^18^. However, also the lateral (in-plane) response can be measured by evaluating the torsion of the AFM cantilever with respect to its long axis and is usually named lateral PFM (LPFM)^19,20^.

Combining VPFM and LPFM potentially provides information on the local orientation of piezoelectric domains in all three spatial dimensions^21^. A full reconstruction needs, however, three independent measurements: one out-of-plane measurement and two in-plane measurements in two independent directions. The combined evaluation of the VPFM and LPFM data is summarized as vector piezoresponse force microscopy (vector-PFM)^22^. Even though PFM is a powerful technique to detect ferroelectric domains on the nanometer scale and is sensitive to local surface displacements down to the sub-picometer regime, it has so far mainly been used to acquire qualitative information. A quantitative vector-PFM measurement is afflicted with a number of difficulties. One major problem is the latter mentioned inherent system background. A detailed description of the most prominent effects can be found in the comprehensive review by E. Soergel^15^. However, in order to reliably reconstruct domain orientation maps/distributions quantitative information is indispensable. That is simply because the magnitudes of the independent VPFM and LPFM measurements in vector-PFM have to be sufficiently accurate to allow for a correct reconstruction of the length and direction of the polarization vectors. Therefore, a proper system calibration and data treatment is necessary. Despite these difficulties, we demonstrate here that one can indeed reconstruct the domain distribution function of a commercial polycrystalline PZT material using vector-PFM. A program based on the software *Mathematica* 10 from *Wolfram Research* ^23^ has been developed to automatically evaluate the measured data sets and display the results graphically.

## Materials and Methods

### Samples

The PFM measurements have been performed on commercial polycrystalline, tetragonal-phase lead zirconate titanate (PZT) ceramics close to the morphotropic phase boundary (MPB), Zr/Ti ratio 50/50, provided by the company PI Keramik (Lederhose, Germany). The selection of a composition close to the MPB is motivated by our purpose to demonstrate the technique on a material relevant for high-performance piezoelectric applications. The corresponding piezoelectric coefficient matrix of the used material in Voigt notation is:^24^

$${d}_{ij}=(\begin{array}{cccccc}0 & 0 & 0 & 0 & \,287 & 0\\ 0 & 0 & 0 & 287 & 0 & 0\\ -97 & -97 & 218 & 0 & 0 & 0\end{array})\,{\rm{pm}}/{\rm{V}}$$

Samples in different poling conditions were investigated: unpoled, in-plane poled, and out-of-plane poled. For electrical grounding, a copper foil tape was attached to the back side of the bulk samples (10 mm × 3 mm × 1 mm) prior to cold embedding. The samples were chemo-mechanically polished with an oxide polishing suspension (OPS) to provide a smooth surface appropriate for AFM/PFM measurements.

### AFM setup

For PFM measurements, an Asylum Research MFP-3D AFM system was employed. The system is equipped with an 80 × 80 µm^2^ × 10 µm closed loop scanner and provides sufficient space under the scanner to conveniently handle samples and the necessary wiring. Even though the system offers a built-in PFM measurement procedure, the applicable voltage range is limited to ±10 V. For poling experiments or to obtain stronger sample response, often higher voltages are necessary. Therefore, the AFM driving voltage signal was read out directly from the AFM controller and fed through a ×10 voltage amplifier *F10A* from *FLC Electronics AB, Partille (SWE)*. The *F10A* can amplify voltages linearly up to a frequency of 1 MHz, which is fully sufficient for standard PFM operation. The amplified driving signal is then put directly to the metal clamp of the AFM cantilever holder with the internal electrical connection to the AFM interrupted. The signal detected via the split photodiode of the AFM feedback system is also read out from the controller and fed into a lock-in amplifier (LIA) (*SR 830* from *Stanford Research, Sunnyvale, CA (USA)*) which is synchronized with the driving voltage frequency. The X- and Y- outputs of the LIA are then fed back to the AFM controller and displayed as separate channels in addition to the topography signal^25^. The external LIA has been used in order to enable access to X-, and Y-signals, and to have full freedom in adjustment of sensitivity, phase, and time constant. Further, it is more reliable to work with the X- and Y-LIA-signals rather than with magnitude (R) and phase (Θ) since those quantities are just recalculated electronically from the primary X-, and Y-signals and thus have a smaller bandwidth^15,26^.

AFM probes used for the PFM measurements were *DCP01* conductive diamond probes from NT-MDT (Moscow, Russia). These probes have a silicon core which is coated with 100 nm of nitrogen doped conductive diamond. The typical tip curvature radius is ~100 nm, and the cantilever’s spring constant is between 2.5 and 10 N/m.

### Measurement procedure

As mentioned above, for vector PFM one out-of-plane and two in-plane measurements are required for a full reconstruction of the polarization orientation. The two in-plane components have to be measured for two independent directions. Practically this means that the sample has physically to be rotated by 90°. After rotation a relocation of the probe to the same area is necessary, which can be rather challenging. Here, the rotation and relocation was performed manually and required additional overview scans in order to find the right location again. In order to facilitate the identification of the same measurement area, regions were preselected that showed distinct surface features like pores or scratches which acted as orientation marks. Initially, 80 µm × 80 µm topography overview scans were recorded at the preselected areas. Within these areas, the final 10 µm × 10 µm areas for inspection were defined. The first two measurements which are one VPFM and one LPFM measurement for the out-of-plane and one in-plane component can conveniently be measured without moving the sample. For the second in-plane measurement, the sample rotation has to be done. The full vector PFM data then contains 6 data sets which are the X-, and Y-LIA data for the three components of piezoresponse in x, y, and z.

### Data evaluation

The theoretical background for quantitative vector PFM has been elaborated in a series of papers^22,27–33^. The theoretical effective piezo-coefficients of tetragonal material (point group 4 mm) “as seen from the tip” in x-, y-, and z-direction can be expressed as^30^:

1a $${d}_{zx}=-({d}_{31}-{d}_{33}+({d}_{15}+{d}_{31}-{d}_{33})\cdot \,\cos (2\cdot \vartheta ))\cdot \,\cos (\phi )\cdot \,\sin (\vartheta )$$

1b $${d}_{zy}=-({d}_{31}-{d}_{33}+({d}_{15}+{d}_{31}-{d}_{33})\cdot \,\cos (2\cdot \vartheta ))\cdot \,\sin (\phi )\cdot \,\sin (\vartheta )$$

1c $${d}_{zz}=({d}_{15}+{d}_{31})\cdot {\sin }^{2}(\vartheta )\cdot \,\cos (\vartheta )+{d}_{33}\cdot {\cos }^{3}(\vartheta )$$

**Figure 1:**
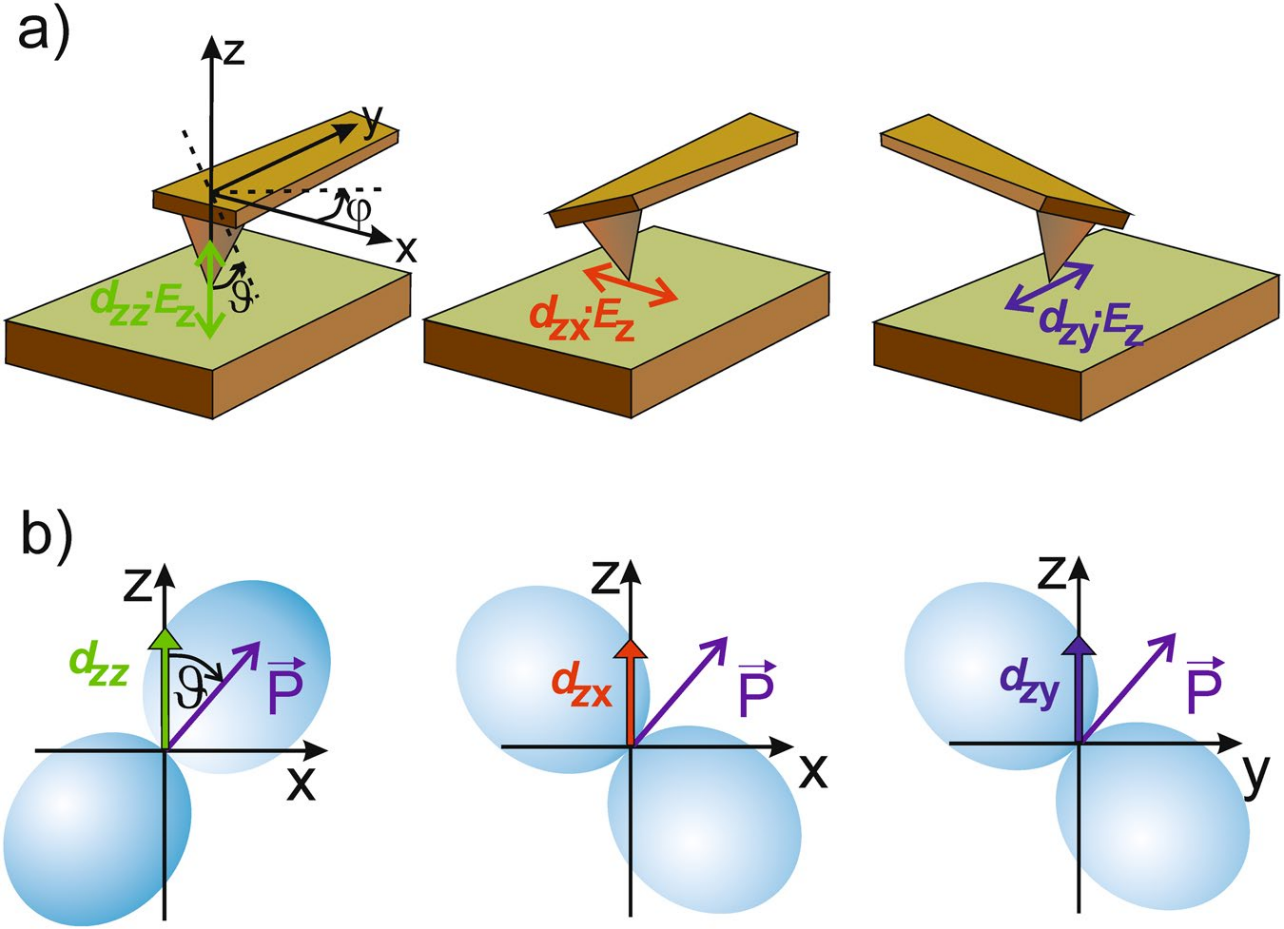
(**a**) Illustration of the laboratory reference system “attached” to the cantilever, and the directions of the corresponding converse piezoelectric displacements induced by the electric field *E*_*z*_ of the tip. (**b**) Schematic of the relation of the piezoelectric surfaces and the polarization vector with respect to the measurement direction. The orientation of the polarization vector has to be varied to simultaneously match *d*_*zz*_, *d*_*zx*_ and *d*_*zy*_ onto the piezoelectric surface.

Here, *φ* and *ϑ* are the Euler angles describing the transformation between the crystal coordinate system and the coordinate system defined by the AFM cantilever (see Fig. 1a). In this case, *ϑ* denotes the angle between the direction of the electric field (surface normal) and the [001] crystallographic axis of the grain under test. Note that because of the tetragonal symmetry only two Euler angles are needed for a description instead of three as for the general case. In our case, we have defined our laboratory coordinate system like indicated in Fig. 1a. Once the displacements (actually, their relative magnitude to each other) and the piezo-coefficients *d*_*ij*_ are known, the relative orientation of the local polarization vector can in principle be reconstructed with the help of the system of Eq. 1a–c. However, Equ. 1 is overdetermined, thus it can only be solved approximatively. Ideally, it would be sufficient to solve only two of the equations, and the third one has to be fulfilled automatically. However, the measured data is by far not as exact as necessary for this approach. Therefore, we use a least-deviation algorithm to find an approximate solution to Equ. 1 that varies *φ, ϑ*, until the best match to the measured data is found. An illustration of the approximation procedure is provided in Fig. 1b. This is performed for each set of corresponding pixels of the measured data (see later). In order to accomplish a data analysis as described above, several data processing steps have to be executed.

**Figure 2:**
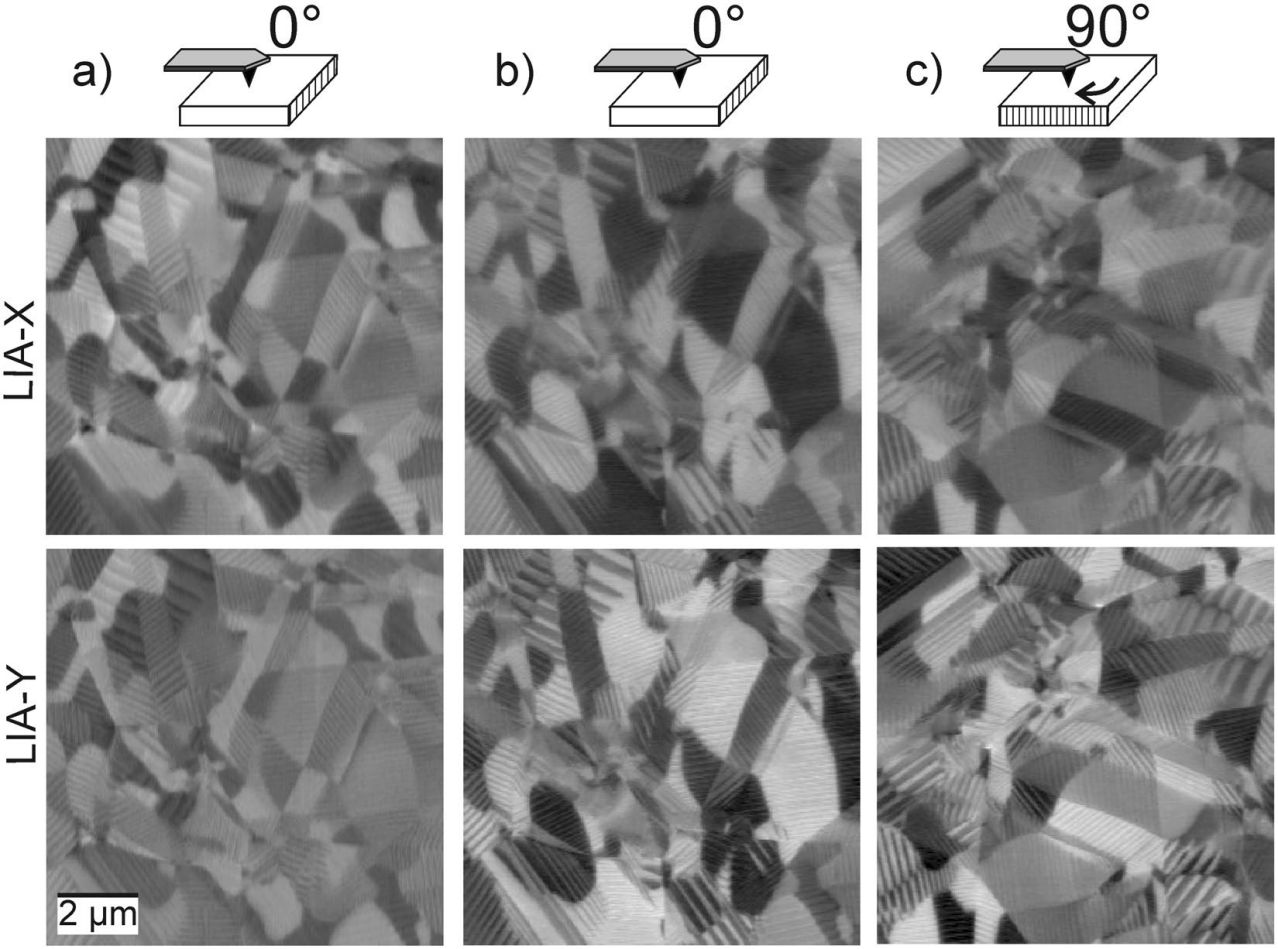
Raw PFM data for X- (top row), and Y- (bottom row) LIA signals obtained for (**a**) VPFM (out-of-plane), (**b**) LPFM in x-direction, and LPFM in y-direction (sample rotated by 90°).

Here, we use the free AFM analysis software *Gwyddion* ^34^ and the commercial software Wolfram Mathematica 10^23^ for data evaluation. Starting point of the evaluation is a set containing topography data as well as X-, and Y-LIA output. A typical set of PFM data obtained from a 10 µm × 10 µm area of an unpoled PZT sample is shown in Fig. 2 (no topography included). There are clearly areas with sizes ranging from several 100 nm to few µm visible containing parallel stripe patterns. The smallest stripes resolvable have a width of ~50 nm and a repetition period of ~100 nm, whereas the largest stripes exhibit widths around 300 to 400 nm and a repetition period of ~500 nm. The stripe patterns arise from neighboring domains with different polarization directions. For PZT, they are usually formed by either 90° or 180° domain boundaries. Note that at this point the vertical and lateral measurements are not directly comparable since the sensitivities of the LIA and the AFM for vertical and lateral response differ significantly. Therefore, further scaling and data processing as explained in the following are necessary. Gwyddion is used for standard data processing of the topography images (step line corrections, mean plane subtraction, etc.). The topography data are of utmost importance since they serve as reference in order to properly match the VPFM and LPFM data. All data files are converted to an ASCII format to allow processing with *Mathematica*. Further parameters transferred to the program are the LIA sensitivities as well as the deflection inverse optical lever sensitivity of the AFM device.

**Figure 3:**
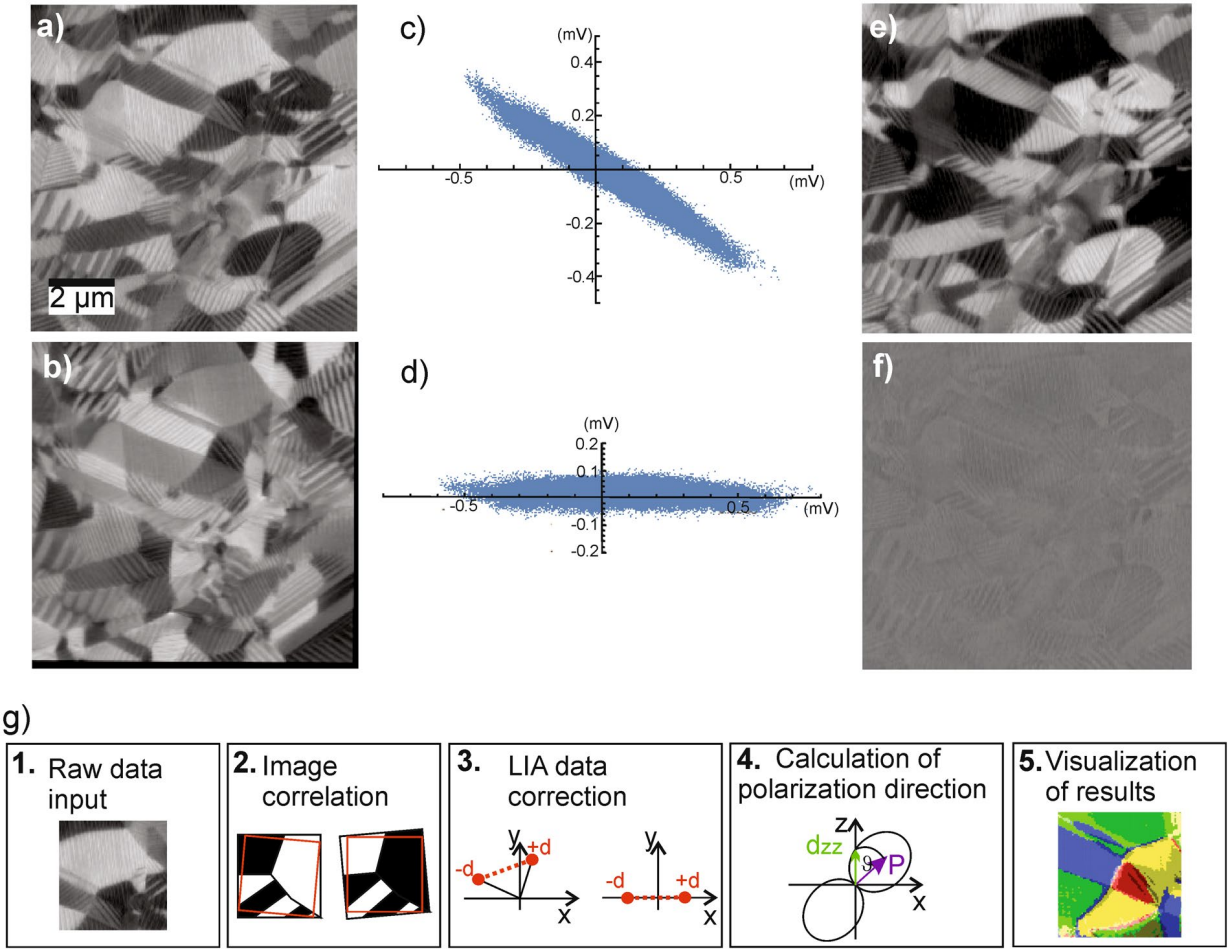
LIA-X signal of the x- (**a**), and y- (**b**) LPFM images shown in Fig. 2 after image matching. The black rim in (**b**) indicates the region where no matching points could be found. The PFM data represented in x-y representation before (**c**) and after (**d**) phase offset and background correction. (**e**) LIA-X signal of the x-LPFM after background subtraction and alignment of the data. (**f**) The LIA-Y data after correction mainly contains noise and almost no image information. (**g**) Illustration of the five main blocks of the data evaluation program.

The first step of the program is importing and converting the AFM data files as needed for further processing. Also the measurement parameters are fed to the program at this point. The second step comprises image correlation and image cropping. It is effectively impossible to obtain a pixel-to-pixel correspondence for the three independent measurements. Thermal drift and incomplete repositioning after sample rotation always cause slight differences in the tip position. In order to find a pixel-to-pixel correspondence, the topography images - recorded simultaneously by the two VPFM measurements of the non-rotated and rotated sample - are compared. One of *Mathematica’s* built-in functions can identify corresponding points in the two topography images. Based on those points a transformation function (rotation and shift) is created and applied to the corresponding X- and Y-data files, respectively. Now all images are aligned such that the corresponding points match. Since the scan areas are usually not exactly the same, there are points (at the image rims) for which no match exists. Therefore, the images are cropped to only the matching areas. Consequently, the image size is reduced depending on how large the overlap for the different measurements was. In Fig. 3a,b, the result after image correlation is presented for the X-LIA data provided in Fig. 2b and c. The thin black rim visible on the right and bottom of Fig. 3b corresponds to points for which no match could be found.

The third part of the program does data correction and evaluates the actual PFM signals for x-, y-, and z-direction. The preprocessed data from the previous step is corrected for the phase offset and the LIA sensitivities. A background correction is done by subtracting the averaged data from independent background measurements for VPFM and LPFM on a glass slide. Basically, the PFM data can be visualized in an x-y graph. Background free, ideal data would just lie on the x-axis. The y-part can be considered as mainly originating from background and noise^15^. In Fig. 3c, an example for background corrected X-, and Y-LIA data in x-y representation is presented. The data scatters considerably and forms a kind of narrow ellipse instead of a line. The tilt of the ellipse’s long axis with respect to the x-axis indicates a phase offset originating from the measurement setup. This offset is corrected by rotating the X-, and Y-LIA data such that the regression line through the data points is parallel to the x-axis (see Fig. 3d). The remaining data scatter in y-direction (width of the data ellipse) can be considered to be only noise. As example, in Fig. 3e the fully correlated, cropped, background, and phase offset corrected X-LIA data derived from the data presented in Fig. 2b is shown. The residual noise in the y-channel can be seen in Fig. 3f. For the further data evaluation only the corrected X-LIA data is used.

The core of the program deduces the solid angles ϑ and φ defining the orientation of the polarization vector of the piezoelectric domain under investigation. Initially, just a qualitative assignment of the polarization vector direction to the octants of a sphere based on the PFM phase is executed. A more precise refinement is then obtained by solving the system of Eq. 1a–c for the input of *d*_*zz*_, *d*_*zx*_, and *d*_*zy*_ derived from the PFM data. An important step is the normalization of the data. Typically, PFM measurements of the same area - even if executed consecutively with no changes of the setup - can vary a little in the magnitude of the obtained signal. Therefore, in general, the three independent measurements (1× VPFM and 2× LPFM) will not perfectly fit together, even though calibration has been done with great care. Thus, data normalization is necessary to obtain correct signal ratios. Here, the data was referenced to a value which was larger than 97.5% of all measured values. That means that all absolute values larger than 97.5% were set to 1 after normalization. After solving Equ. 1, a list is obtained containing the values for the solid angles for each pixel ranging from 0–90° for ϑ and 0–360° for φ.

The final part of the program is creating graphical visualizations of the results, which is an orientation distribution function (ODF) (in multiples of random distribution) of the polarization vector orientation showing the statistical distribution of orientation directions. Further a representation of the scanned area with each pixel colored according to the local polarization vector orientation is generated providing insight into the spatial distribution of the domain orientations. A graphical summary of the major program steps is depicted in Fig. 3g.

### Data availability

The datasets generated during and/or analysed during the current study are available from the corresponding author on reasonable request.

## Results

In the following, we present the results obtained for differently poled PZT samples in order to validate the evaluation program. The poling conditions under consideration are samples with local out-of-plane poling realized by AFM manipulation, samples with macroscopic out-of-plane and in-plane poling, as well as unpoled samples.

### Locally out-of-plane poled PZT sample

**Figure 4:**
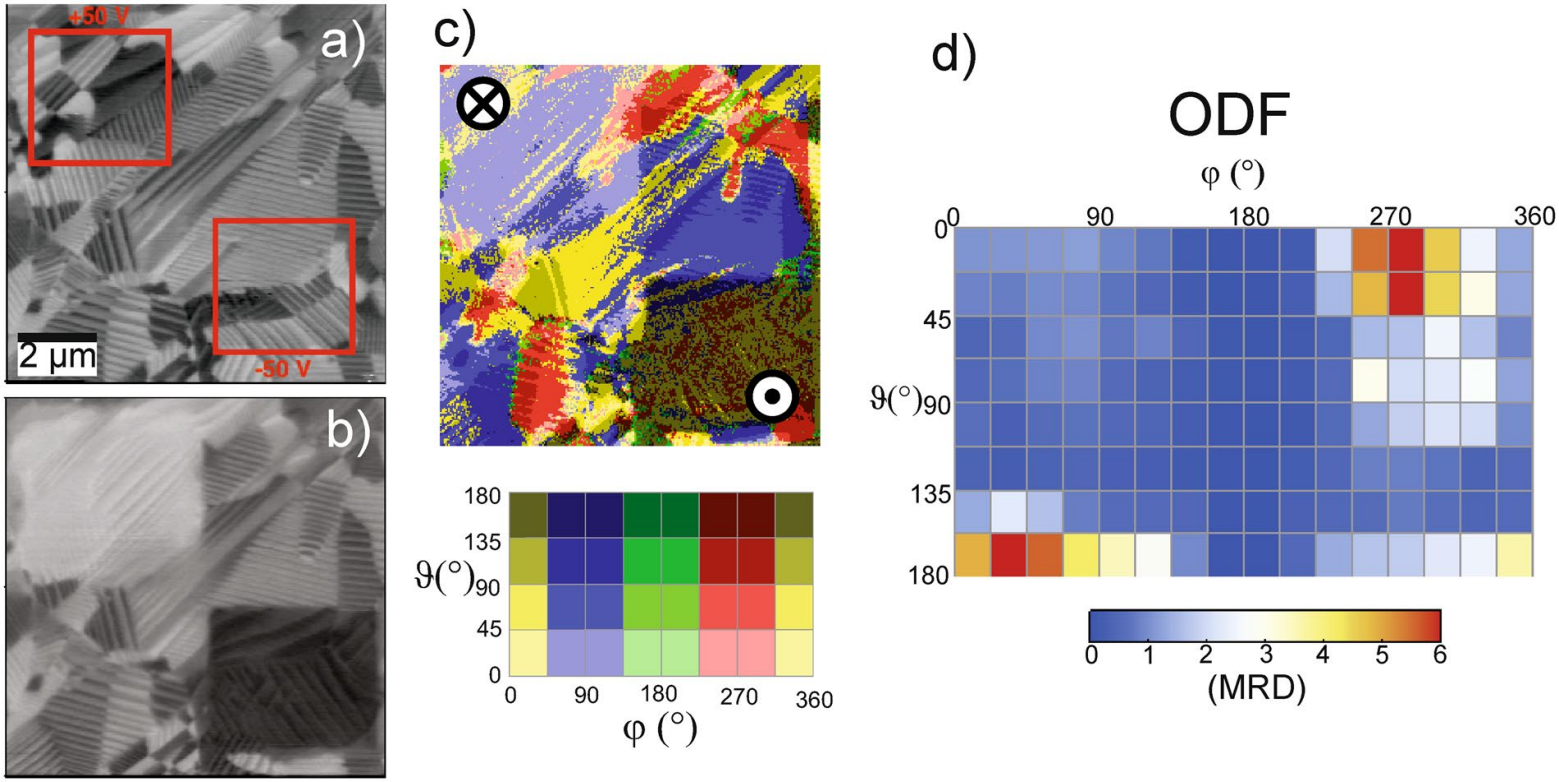
(**a**) X-LIA signal of an unpoled PZT sample. The red squares indicate the positions for the local poling with the corresponding tip bias noted. (**b**) The same area as a) after poling. The clear square changes in contrast indicate successful rotation of the polarization direction. (**c**) Visualization of the polarization vector direction after local poling. The out-of-plane poled areas clearly appear as brighter (ϑ = 0°; polarization pointing downward, **⊗**) and darker (ϑ = 180°; polarization pointing upward, **◉**) contrast. (**d**) The corresponding orientation distribution function (ODF) in multiples of random distribution (MRD) clearly shows accumulation at ϑ = 0° and 180° respectively, which is consistent with out-of-plane poling.

In Fig. 4, the results for a locally out-of-plane poled sample (using AFM manipulation) are shown. Initially, the sample was unpoled, showing domains with parallel lamellar structures within grains, which are well visible in the X-LIA (10 µm × 10 µm) data presented in Fig. 4a. The inspected area contains large grains with diameters between 1.5 and 8 µm. Within the grains, areas with parallel stripe patterns are well visible. The regions with uniform stripe patterns can be as small as only 400 nm but can also extend to about 4 µm. The minimal stripe period found is about 120 nm whereas the biggest is three times larger. Two 2.5 × 2.5 µm^2^ square regions - as indicated by the red squares in Fig. 4a - have been selected within this area for poling. The poling has been performed by scanning the selected areas with a DC-biased AFM tip (contact force ~100 nN). For the upper left area in Fig. 4a, a bias of +50 V and for the bottom right area −50 V have been chosen. As can be seen in Fig. 4b, the successful poling manifests itself by significant contrast changes in the square-shaped poled regions. Apparently, the poling created new domain structures. Stripe direction, width, and period have clearly changed in the poled regions. In general, the stripe width and period have increased. The largest stripe period of ~600 nm is observable in the square poled at −50 V (dark square region in the bottom right of Fig. 4b). In addition, the new stripe patterns generated by AFM poling are less ordered than the pristine ones. In Fig. 4c, the illustration of the local polarization directions clearly shows that the individual lamellas appear to be domains of a uniform polarization direction. Further, an accumulation of ϑ values around 0° and 180° respectively is visible in the poled regions. Accordingly, the ODF (Fig. 4d) also exhibits an increased number of ϑ-values between 0° and 45° (polarization pointing downward) and 135° and 180° (polarization pointing upward) which is fully consistent with what one would expect from out-of-plane poling.

For the sake of completeness, the experiment has been repeated at a different position but with a reduced DC-bias of only ±25 V (not shown). Qualitatively, the results were identical with those obtained on the samples poled with ±50 V. However, in the latter case the ODF shows a less pronounced alignment of the domains as is expected for the weaker field, and thus this result  is also consistent with the expectations.

### Macroscopically out-of-plane poled PZT sample

**Figure 5:**
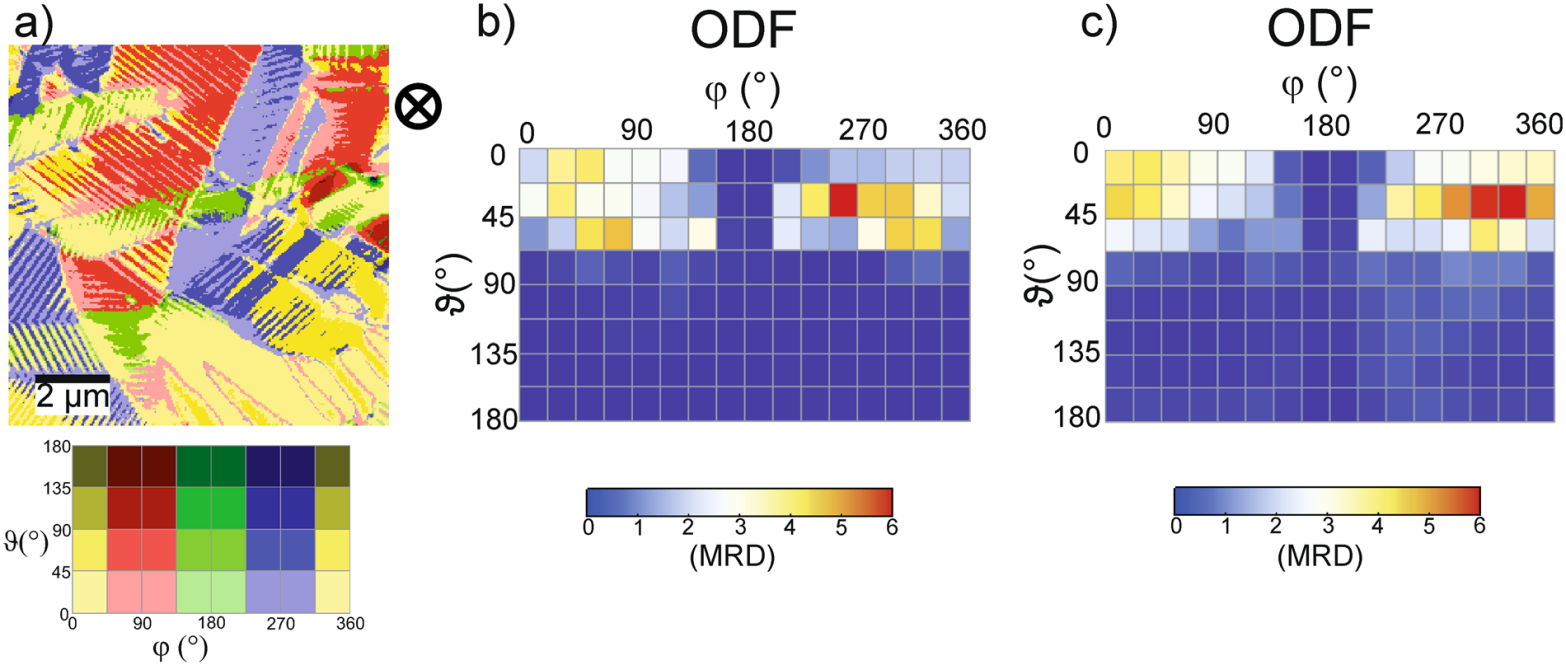
(**a**) Visualization of the polarization vector directions of a macroscopically out-of-plane poled PZT sample in color coded representation. (**b**) Orientation distribution function according to (**a**). (**c**) Average orientation distribution function of seven independent measurements at different positions of the out-of-plane poled sample.

In comparison to the locally out-of-plane poled samples also macroscopically poled samples have been investigated. Such samples have been prepared by application of a strong electric field during the production process. Here, the PFM images are also dominated by lamellar ordered stripe domains arranged in few µm large grains (see Fig. 5a). The grains are typically elongated with lengths between 2 and 5 µm and widths of 1 to 2.5 µm. Typically, the stripes are roughly oriented perpendicular to the grains’ long axes. The stripe domains usually exhibit lengths from 1 to 2.5 µm and stripe widths of about 200 nm. Even though few grains appear uniform (without stripes), the majority of the grains are stripy where adjacent stripe domains differ significantly in ϑ and/or φ. In Fig. 5b, the corresponding ODF is shown. Noticeably, there is a strong imbalance between ϑ = 0° and 180° orientations. The lack of 180° domains indicates that the majority of the polarization vectors point downward, as can be expected for an out-of-plane poled sample. The evident absence of counts around φ = 0° and 180° is an apparent artefact of the evaluation procedure. We think it arises from the fact that the LPFM values are in general very small (close to zero). The least deviation algorithm then mostly “finds” different directions for angles close to 0° and 180°.

### Macroscopically in-plane poled PZT sample

**Figure 6:**
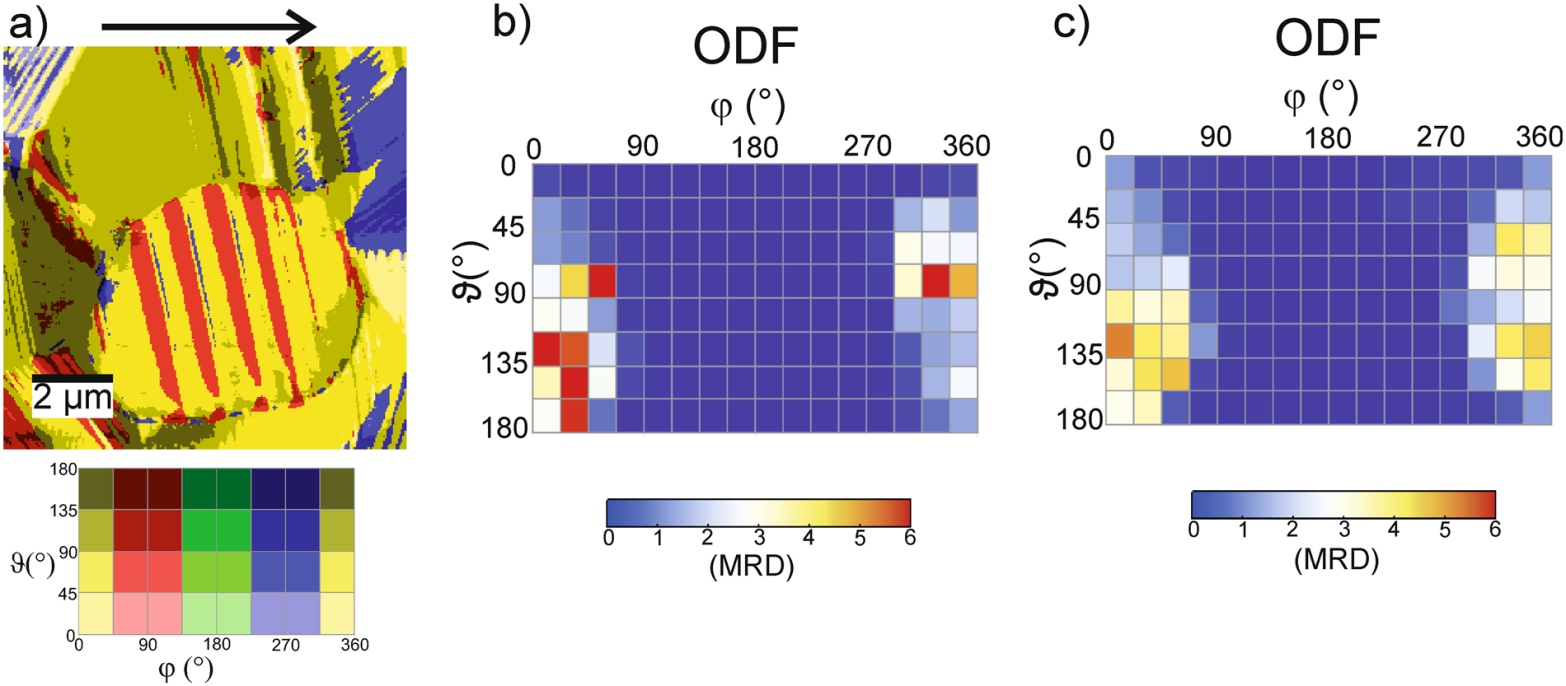
(**a**) Color-coded representation of the local domain orientation of an in-plane poled PZT sample (poling direction indicated by the black arrow). (**b**) Orientation distribution function derived from (**a**). (**c**) ODF averaged from seven independent measurements in different places on the in-plane poled PZT sample.

Analogously to out-of-plane poled samples, in-plane poled samples have been prepared. Figure 6 shows an illustration of the local polarization directions of an in-plane poled sample (poling direction from left to right). The area probed in the in-plane sample has larger grains (lateral size of about 6 µm) than the one probed in the out-of-plane poled samples; the lengths of the stripe domains range here from 1.5 µm to about 4 µm. Correspondingly, the width of the stripes usually scales with their length. Thus, the longer stripes can reach widths of ~500 nm whereas the short ones exhibit stripe widths around 150 nm (compare to Fig. 6a). However, there are also areas as large as 2.5 µm without stripe domains. These are either large areas of uniform polarization or areas where the stripe structure could possibly not be resolved. The ODF presented in Fig. 6b corresponds to the data provided in Fig. 6a. Clearly, there is a massive lack of orientations with φ values between 90° and 270° whereas a wider angle range in ϑ is present. That means that no in-plane components oriented between 90° and 270° are present, which indicates a pronounced in-plane texture. The polarization vectors aligned in plane still possess components with all possible out of plane-orientations as indicated by the wide range of ϑ. Also, averaged data over seven independent measurements on the same sample, but at different positions reflect this behavior (see Fig. 6c). This is exactly what one would expect from preferential in-plane orientation of the domains.

### Unpoled samples

**Figure 7:**
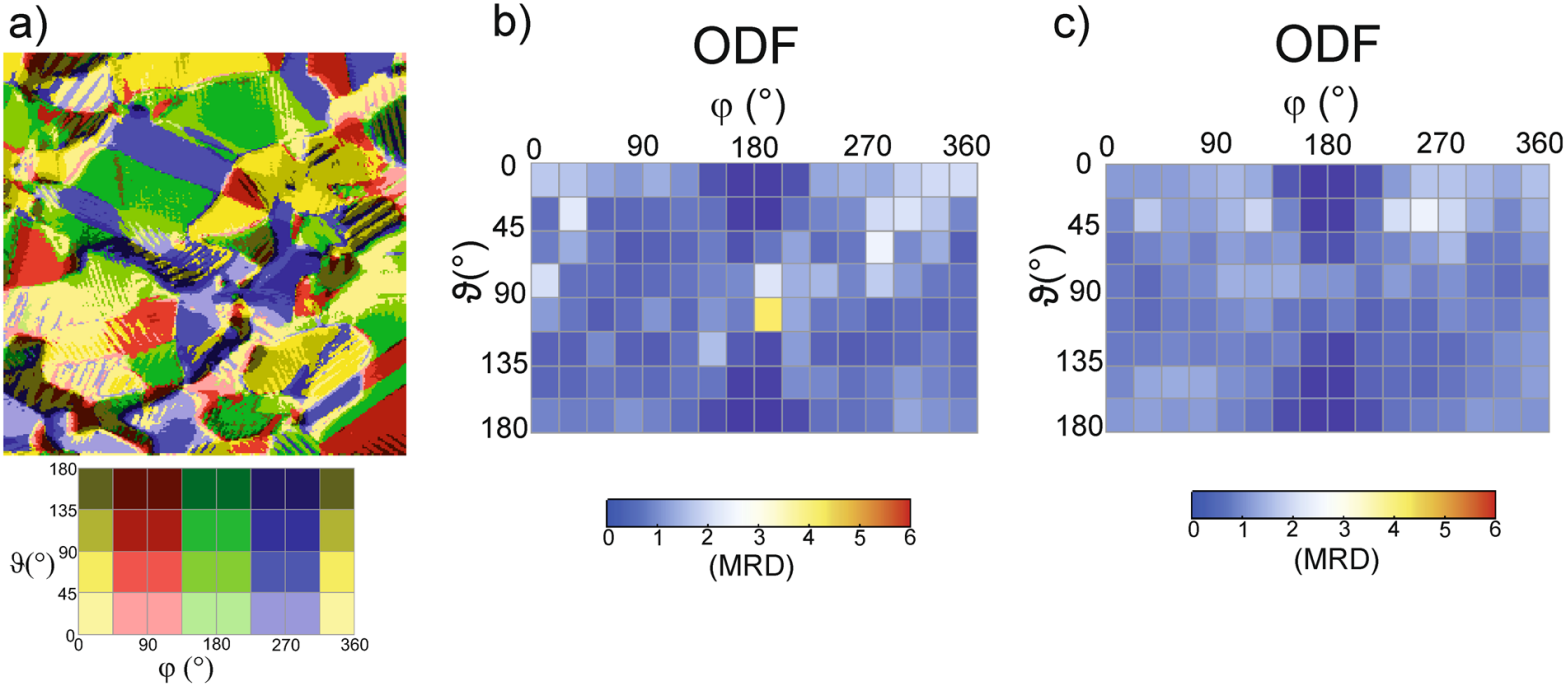
(**a**) Color-coded illustration of the local domain orientation of an unpoled PZT sample. (**b**) Orientation distribution function derived from (**a**). (**c**) ODF averaged over seven independent measurements in different places on the unpoled PZT sample.

Finally, nominally unpoled PZT samples were investigated. The results are compiled in Fig. 7. Similar to the poled samples, pronounced lamellar domain structures are visible. The grain size in the area investigated here is 3 µm on average, but grains as small as 1.5 µm are also present. The lengths of the stripe domains are usually only limited by the lateral grain size and typically range from 0.5 µm to 3 µm. The corresponding stripe widths lie between 100 nm and 250 nm. In Fig. 7a, the color coded map of the local polarization is depicted. The corresponding ODF is provided in Fig. 7b showing no preferential domain orientation. A very similar situation has been found on six other places on the same sample. The averaged ODF over seven independent measurements is presented in Fig. 7c and is consistent with the expectations for an unpoled sample, in which no distinct polarization direction is present.

## Discussion

Overall, the deduced ODFs (Figs. 4– 7) obtained for the different PZT samples agree well with the intuitive expectations for the differently poled samples. However, as pointed out by Kalinin *et al*.,^22^ for a full reconstruction the number of possible orientation directions has to be limited and known. Even though the knowledge of the crystallographic structure of a single crystalline material is sufficient to reconstruct the polarization orientation, this task is much harder to accomplish for materials with random grain orientation. For example, Munoz-Saldana *et al*. selected only grains with {001} crystallographic direction within polycrystalline PZT by identification and recognition of square-net structures in the etch patterns^35^. Roelofs *et al*. measured nanoscale in-plane and out-of-plane hysteresis loops and monitored the signal changes upon domain switching to reconstruct the three-dimensional polarization distribution of individual grains^36^. A three-dimensional polarization domain reconstruction based on VPFM and LPFM has been realized for (111)-oriented PZT capacitors^37^ and ZnO thin films with a limited number of orientation possibilities^38^. In the tetragonal phase of PZT, six equivalent polarization directions exist, corresponding to the [100], [−110], [010], [0–10], [001], and [00–1] directions of the para-electric state. In our case, we deal with a polycrystalline bulk material that statistically exhibits all possible orientations of the grains. Within the grains, there are several domains with different polarization directions. Therefore, we have no general crystallographic reference frame to which the measured piezoresponses can be correlated. The measurable signal for each grain is basically the projection of the piezoelectric surface onto the plane which is parallel to the real surface^37^. For example, in ref.^38^ the textured ZnO film exhibited only 4 different orientations of the ZnO grains which should provide 4 different levels in the vertical PFM signal, thus, an attribution of crystallography and polarization was relatively simple.

**Figure 8:**
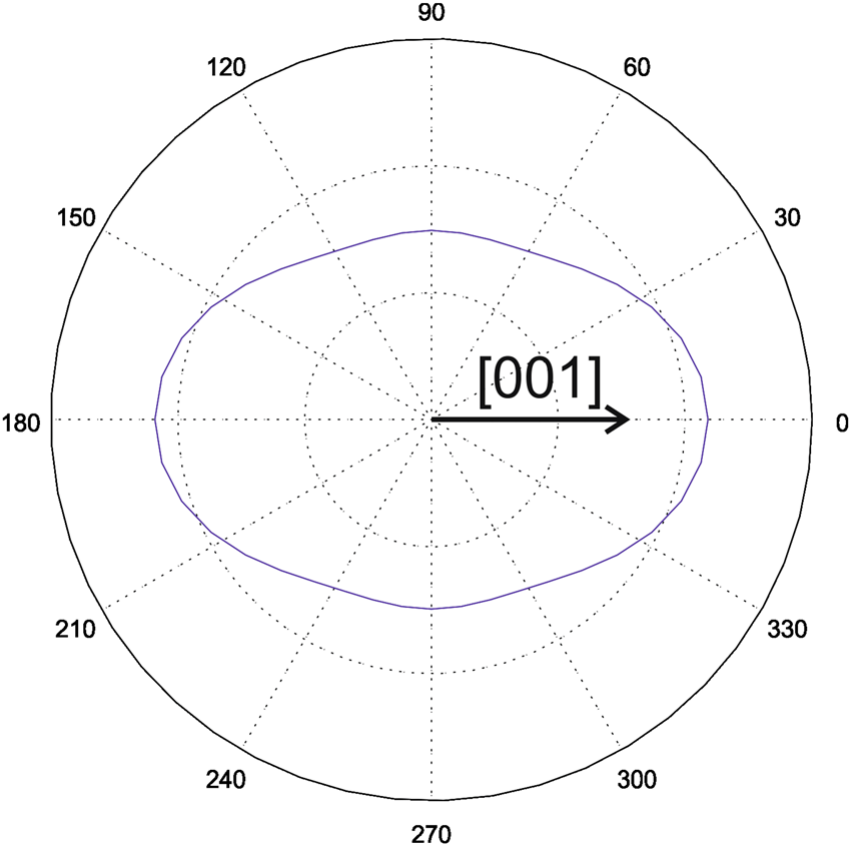
Piezoresponse of tetragonal PZT as a function of measurement angle with respect to the polarization vector lying in [001] direction.

In our case, responses can vary continuously between the expected minimum and maximum values. However, at least the domains within a single grain with only one crystal orientation should provide correct relative responses. In Fig. 5a, for a macroscopically out-of-plane poled sample, a number of grains with stripe-like domains are visible. Comparison with the added color code for the orientation angles reveals that the polarization directions of adjacent domains are either rotated by 90° or 180°. A 90° rotation of the in-plane polarization between neighboring domains is well visible on the single ellipsoidal grain in the center of Fig. 6a. 90° and 180° domain walls are the expected domain structure in tetragonal PZT material. Thus, at least within single grains the evaluation procedure seems to provide the correct results. An overall reference frame - linking the results of the individual grains - is defined by the maximum responses measured on the inspected area. We know from the preparation process that statistically all possible grain orientations should be present. Typically, we find a large number of domains within the inspected area. In Fig. 8, the theoretically expected responses are provided as a function of measurement direction with respect to the polarization vector lying in [001] direction. We now just assume that the large set of domains measured contains also some which are oriented to yield the maximum/minimum possible response. Maximum response is expected for a grain with its (001) plane parallel to the surface, such that the measuring direction is parallel or antiparallel to the polarization vector. Minimum response is expected for a grain with the (001) plane tilted by ~77° towards the surface (see Fig. 8). This also implies a certain uncertainty because there is no guarantee that the maximum/minimum response domain is really included in the measured area. However, - under the condition that there is a sufficient number of domains accessible - at least domains oriented close to the maximum/minimum condition should be present. Since we set all values within the top 2.5% and lowest 2.5% to maximum and minimum, respectively, an error of at least that order of magnitude is inherent. However, we assume that the error originating from calibration - even though performed as accurately as possible - is usually larger than that.

Further, we assume dielectric isotropy, which in general is incorrect. Of course, polycrystalline PZT material macroscopically behaves dielectrically isotropic. However, a single crystal exhibits a dielectric constant which in general depends on the crystallographic orientation, and hence the electric field distribution generated by the biased tip also depends on the orientation. As pointed out by Eliseev *et al*.^31^, assuming dielectric and elastic isotropy is usually well justified for ferroelectric perovskites. A typical value for the dielectric constants in two major directions of a tetragonal PZT material are ε_33_ ~ 1200 and ε_11_ ~ 1130^39^. Therefore, the dielectric anisotropy defined as γ = (ε_33_/ε_11_)^1/2^ is close to unity. Hence, we assume that this effect is negligible compared to other sources of error. The effective dielectric constant is (ε_33_ · ε_11_)^1/2^ = 1164 for PZT, which means that the effective penetration depth of the electric field is very limited. Typically, the electric field reduces to 1% of its value at the surface within ~1 µm towards the bulk. Since the grain size is in the range of a few µm, a contribution to the signal from the material surrounding the grain under test is unlikely.

An additional point that has to be considered is the homogeneity of the material. As mentioned, we chose to demonstrate the technique on an application-relevant material and thus selected a tetragonal PZT composition close to the morphotropic phase boundary (MPB). Due to MPB proximity, the material might contain grains of rhombohedral symmetry as well^40^. In this case, there also exist grains for which a different piezoelectric coefficient matrix applies which leads to wrong results at least for the individual rhombohedral grain. In the worst case, a rhombohedral grain can affect the total evaluation in case it exhibits the maximum/minimum response, which is used to define the extreme positions of the polarization vector. However, statistically less than 30% of the grains are expected to be rhombohedral in the investigated composition^40^. We therefore consider it as a minor source of error. On the other hand, a major source of potential errors is the calibration. Here, especially the calibration of the lateral response is critical as already discussed in the experimental section. An improvement in the quality of calibration can be obtained if a calibration device - like for example has been employed in the group of E. Soergel - is utilized^41^.

## Conclusion

Based on a set of commercial PZT samples the challenge was tackled to reconstruct the orientation distribution function (ODF) of the spontaneous polarization in polycrystalline tetragonal PZT material using vector piezoresponse force microscopy (vector-PFM). For ODF reconstruction, a *Mathematica* code has been developed that automatically processes vector PFM data (one vertical and two in-plane measurements) and delivers the ODF and a map of the local polarization directions. Within individual grains that spanned a size range from 0.5 µm to 6 µm, domain patterns consisting of parallel stripes were the dominating features of all samples. The lengths of the stripe domains often extended over the whole grain diameter with stripe widths ranging from 150 nm to 600 nm. The for PZT expected 90° and 180° rotation of the polarizations direction of adjacent domains is well resolved by the evaluation method. Local poling of originally unpoled PZT achieved by scanning a ±50 V biased AFM tip across a predefined area resulted in a clear out-of-plane polarization and completely different domain structures compared to the pristine sample. The poling results were satisfactorily tracked by the *Mathematica* based data evaluation algorithm and agree with the expected behavior. In addition, PZT samples macroscopically in-plane and out-of-plane poled were investigated. The obtained ODF and the map of the polarization directions are well in accord with the expectations, suggesting the validity of the approach. We are confident that the developed tool will be very helpful for the analysis and deeper understanding of the material’s behavior in PZT devices. Especially, the influence of highly localized phenomena like mechanic stress, cracks or highly anisotropic electric fields in the vicinity of electrodes, etc. that might appear in devices can be studied in detail in the future.
